# Supplementary figures and images for: Quantitative perturbation-phenotype maps reveal nonlinear responses underlying robustness of PAR-dependent asymmetric cell division
Source: PLoS Biol. 2024 Dec 9;22(12):e3002437. doi: 10.1371/journal.pbio.3002437 (PMC11627365; doi:10.1371/journal.pbio.3002437)

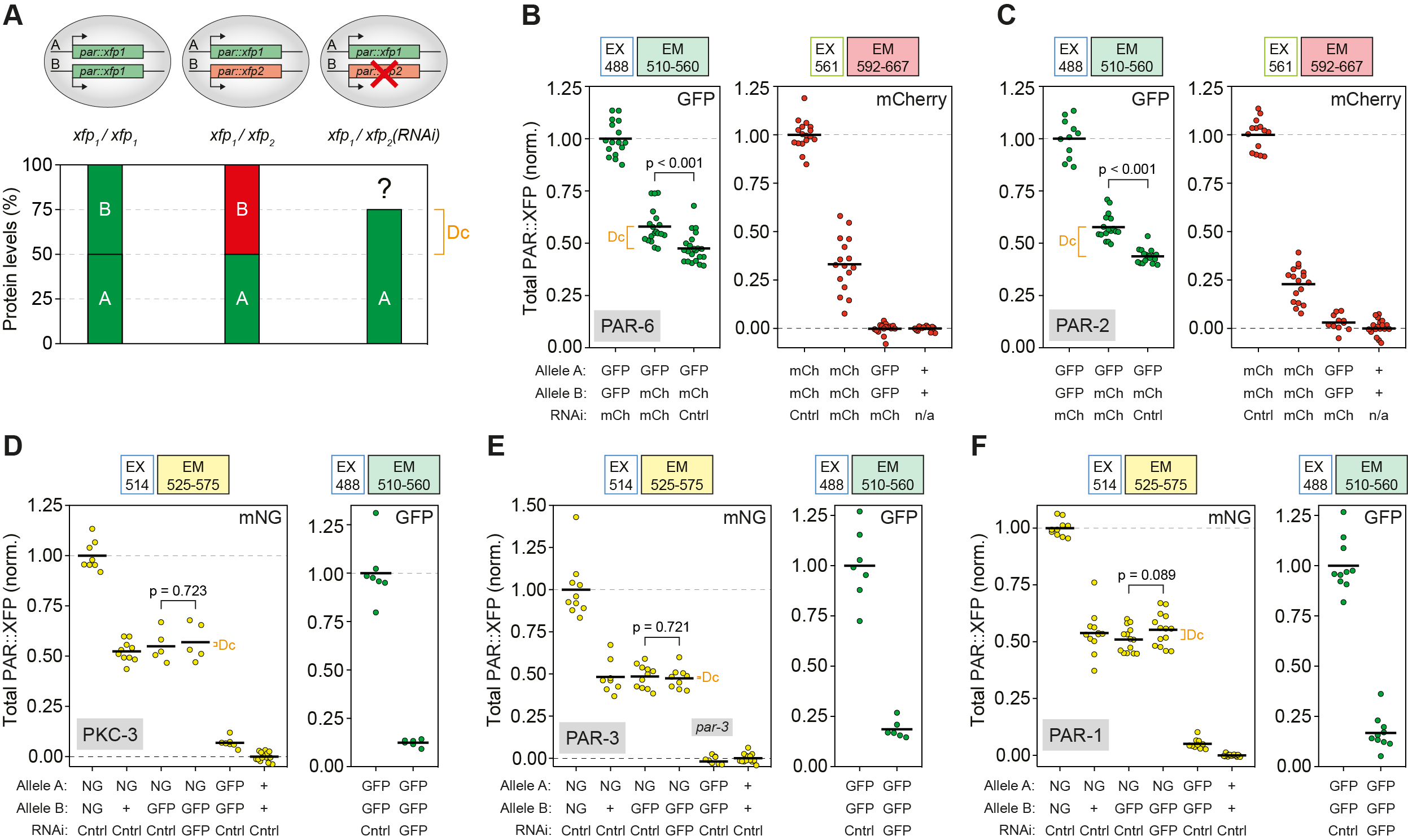

Supplement: S1 Fig — (A) Schematic for dosage compensation assay using allele-specific RNAi-depletion. (B, C) PAR-6::GFP (B) and GFP::PAR-2 (C) levels for gfp/gfp, gfp/mCherry, and gfp/mCherry(RNAi) genotypes (left) together with controls for the depletion of mCherry-tagged alleles by RNAi (right). Note that compensation, if present, should be manifest as a difference in GFP levels in gfp/mCherry embryos ± mCherry(RNAi). gfp/gfp and +/+ embryos are shown to control for bleedthrough into the mCherry channel and to confirm zero point, respectively. (D–F) mNG::PKC-3 (D), mNG::PAR-3 (E), PAR-1::mNG (F) levels for the indicated genotypes (left) together with controls for depletion of GFP-tagged alleles by RNAi (right). Note that compensation, if present, should be manifest as a difference in mNG levels in mNG/gfp embryos ± gfp(RNAi). Statistics, unpaired t test. gfp/gfp and +/+ embryos are shown as controls for specificity of mNG excitation and to confirm zero point, respectively. The raw data underlying this figure can be found at https://doi.org/10.25418/crick.27153459. (TIF) [file pbio.3002437.s001.tif]

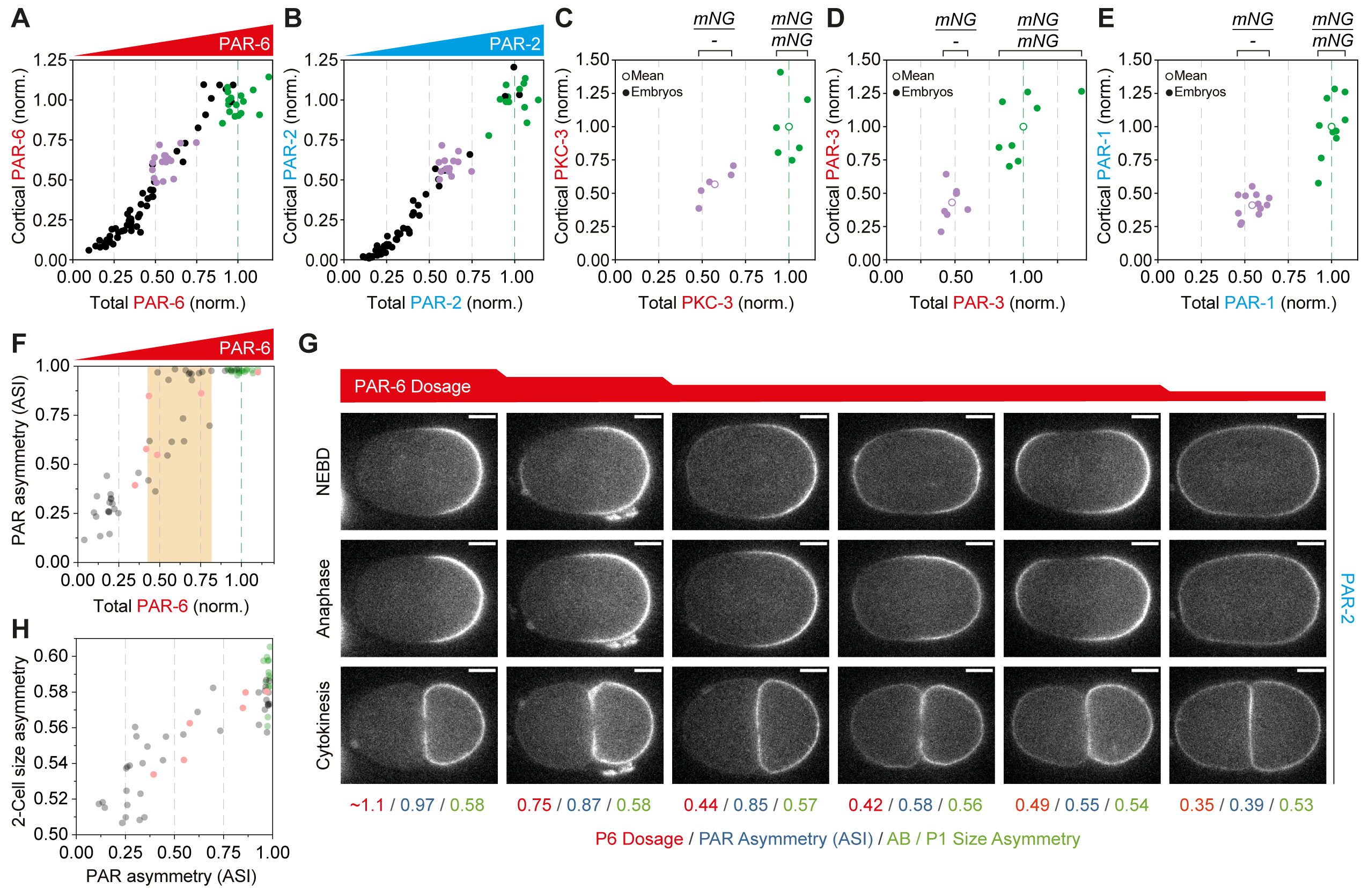

Supplement: S2 Fig — (A, B) Cortical concentrations of PAR-6::GFP(A, NWG0119) and GFP::PAR-2(B, NWG0167) decline as a function of total dosage. (C–E) Cortical concentrations of PKC-3(C), PAR-3(D), and PAR-1(E) are reduced approximately 50% in heterozygous (mNG/-) embryos relative to homozygous (mNG/mNG) controls. Colored data points in (A–E) indicate homozygous (xfp/xfp, green) and heterozygous (xfp/-, purple) embryos. Note heterozygous conditions in (A–E) were par-X(xfp1/xfp2(RNAi)) as in Fig 2D. (F) PAR asymmetry (ASI) as a function of total PAR-6 dosage as in Fig 4E, but highlighting individual embryos shown in (G). (G) Example embryos showing PAR-2 localization at NEBD, anaphase and cytokinesis for differing PAR-6 dosage. Dosage, PAR asymmetry, and size asymmetry are shown below each embryo. Embryos shown in (G) are depicted as red data points in (F, H). (H) Two-cell size asymmetry as a function of PAR asymmetry. Scale bars, 10 μm. The raw data underlying this figure can be found at https://doi.org/10.25418/crick.27153459. (TIF) [file pbio.3002437.s002.tif]
