# Supplementary material for: Quantitative perturbation-phenotype maps reveal nonlinear responses underlying robustness of PAR-dependent asymmetric cell division
Source: PLoS Biol. 2024 Dec 9;22(12):e3002437. doi: 10.1371/journal.pbio.3002437 (PMC11627365; doi:10.1371/journal.pbio.3002437)
Supplement: Attachment — Submitted filename: PBIOLOGY-D-23-02880R2_Response2Reviewers.pdf [file pbio.3002437.s004.pdf]

Response to Comments - Rodrigues et al.

Thank you for your patience while your manuscript entitled "Nonlinear readout of spatial cues underlies robustness of asymmetric cell division" went through peer-review at PLOS Biology and please accept my apologies again for the delay in providing you with our decision. Your manuscript has been evaluated by the PLOS Biology editors, an Academic Editor with relevant expertise, and by two independent reviewers.

The reviews are attached below. As you will see, the reviewers find the conclusions interesting, but they also raise several concerns that should be addressed to consider the manuscript for publication. Reviewer 1 asks for a couple of clarifications regarding the phenotype observed when PAR-2 is depleted, and also misses an analysis of the anterior and posterior pulling forces in the experiments on spindle positioning that could inform on the robustness of the spindle positioning pathway. This reviewer also would like further discussion taking into consideration the field of modelling PAR polarity in the C. elegans embryo, and questions to what extend the current models predict that changes in PAR protein level will lead to the observed effects of reducing cortical levels while maintaining overall polarisation. Reviewer 2 would also like you to clarify several points about the data and the way some of the experiments were performed, some of the terms used, and the meaning of several statements included in the results, among other issues.

In light of the reviews, we are pleased to offer you the opportunity to address the comments from the reviewers in a revision that we anticipate should not take you very long. We will then assess your revised manuscript and your response to the reviewers' comments with our Academic Editor aiming to avoid further rounds of peer-review, although might need to consult with the reviewers, depending on the nature of the revisions.

Thank you for your enthusiasm and to the reviewers for the constructive comments on our manuscript. In response to these comments we have undertaken a number of improvements:

(1) We have now performed spindle severing assays in heterozygotes as requested by Reviewer 1. These experiments revealed no detectable difference between wild-type and heterozygous embryos, suggesting forces acting along the A-P axis are not detectably changed. These are now included in Figure 6 and corresponding text added.
(2) We have rewritten several sections and added additional discussion, which we hope addresses the concerns of the two Reviewers regarding clarity of the text.

We apologize that it has taken longer than expected to submit our revision and hope it now meets your approval. Detailed responses are included below.

Reviewers' comments

Rev. 1:
In this manuscript the authors investigate to what extent PAR-dependent polarity establishment (PAR polarity itself and downstream size asymmetry and spindle positioning) in the one cell embryo of C. elegans is robust to changes in protein dosage, and what the sources of robustness are. The work is based on prior observations in the field that polarization is resilient to changes in temperature, pressure on the embryo, gene dosage, and symmetry breaking cues.

The manuscript begins by examining whether robustness to changes in par gene dosage are due to compensatory dosage regulation. The experiments are performed thoroughly and investigate 5 different par genes, making use of a recently optimized fluorescence quantification protocol also developed in the Goehring lab. While some dosage compensation is observed, protein levels in heterozygous animals as severely reduced, indicating that dosage compensation is not a major driver of robustness. Downstream readouts of PAR polarity, specifically division size asymmetry and spindle positioning, are also robust to changes in PAR protein dosage.

To investigate the source of robustness, the authors quantitatively measure cortical PAR protein levels and distribution upon reduction of overall PAR protein levels. Overall, while cortical PAR concentrations are affected, polarized PAR domains are observed until PAR protein levels are depleted below a threshold value, indicating that PAR polarity establishment itself is robust to changes in PAR protein levels. This raises interesting questions on how downstream pathways read out PAR polarity.

Finally, the authors investigate the interplay in robustness between symmetry breaking cues (which similarly are robust to perturbations) and PAR polarity. They find that changes in either system that by themselves are tolerated,

sensitize to changes in the other system. E.g., when cortical flow is reduced, changes in PAR dosage that are normally tolerated now result in failure to form polarized PAR domains.

Overall I find all of the experiments very rigorously performed and well documented. The only potential cause for concern for acceptance in the flagship PLOS journal is that there are no mechanistic insights into how downstream pathways robustly translate PAR polarity into asymmetric division. However, this study provides a strong and necessary foundation for such studies.

I have a few smaller points that I think need clarification.

In figure 4 the authors claim that 'When PAR-2 is depleted, the anterior PAR-6 domain expands into the posterior.' Looking at panels C and D, I can't see this. The boundary of the PAR-6 domain seems to stay the same regardless of the levels of PAR-2.

We agree that the changes are modest and much weaker than the invasion of PAR-2 in the converse experiment, which is likely due to additional stabilizing feedback absent in prior models (see below for details). That said, there is a statistically significant, albeit modest, extension into the posterior, which we now explicitly quantify (New Figure 5D). We would note that this is consistent with prior work that PAR-6 generally remains asymmetric in *par-2(RNAi)* at these timepoints - *e.g. in 10.1126/science.1208619, par-2 hets* result in only a 10% increase in domain size (~0.5 to ~0.55), while *par-2(RNAi)* increases fractional PAR-6 domain size by 50% (~0.5 to ~0.75). We have tried to make this more clear in the text.

Given this resilience of PAR-6 asymmetry to PAR-2 depletion, it is striking that asymmetric division appears to respond in a quantitatively similar manner to depletion of either PAR-6 or PAR-2, suggesting that the magnitude of aPAR asymmetry alone is not a good predictor of successful asymmetric division. Thus it seems unlikely to be as simple as PKC-3 levels defining the profile of force generator activity. We have now noted this in the text.

In the experiments probing the spindle positioning pathway, I was expecting to see an analysis of the anterior and posterior pulling forces, a relatively standard approach in the field that directly analyzes forces acting on the spindle rather than relying on secondary readouts like oscillations. Performing this analysis would better inform on whether the robustness of the spindle positioning pathway is at the level of force generation or not. Perhaps the finding that oscillations are not perturbed in par-6(+/-) is due to the relative insensitivity of the assay.

We have now performed spindle severing experiments in heterozygotes for comparison and observe no significant difference in spindle pole behavior relative to wild type, suggesting minimal changes in the forces acting along the A-P axis.

I was hoping for a more detailed discussion placing the findings of this manuscript into the broader field of modeling PAR polarity in the C. elegans embryo. To what extent do current models, including those of the senior author himself, predict that changes in PAR protein level will lead to the observed effects of reducing cortical levels while maintaining overall polarization.

The authors write that "Specifically, we predict that polarity outputs in otherwise wild-type embryos should be robust to changes in cue strength and, conversely, that reductions in cue strength should render polarity outputs sensitive to PAR dosage". I was puzzled when comparing this with modeling and observations in the 2011 Science publication of the senior author.

Quoting from that paper on expectations of the model: "changing the amount of a given PAR protein should result in changes in the steady-state position of the PAR boundary (fig. S6). We found that PAR-2 domains were larger in embryos possessing only one functional par-6 gene copy (Fig. 4, D and E) and in embryos where PAR-2 was overexpressed by optimizing the codon adaptation index (CAI) of the green fluorescent protein (gfp)::par-2 transgene (Fig. 4, F to H)." In the current manuscript, embryos possessing only one par-6 copy do not appear to have an altered PAR boundary. How should I reconcile these seemingly conflicting observations, and what does this say about our current PAR polarity models?

There are several points to unpack here:

(1) **Impact of PAR dosage reductions on PAR boundaries** - In Goehring et al 2011, the experimental trends for PAR-6 reduction are similar to the data here: PAR-2 occupies a larger fraction of the membrane (either a larger posterior domain or formation of a second anterior domain) when PAR-6 levels were reduced by RNAi or in *par-6(hets)*. As noted above, the impact of PAR-2 reductions on PAR-6 domains are much more modest than would be expected for a simple reciprocal feedback model; however, this may be partially explained by

the existence of additional aPAR stabilizing circuits, such as the CDC-42 GAP CHIN-1, PAR-3 oligomerization, and maintenance phase cortical flows. While the general qualitative trend linking relative dosage and domain size holds, we also generally don't observe clear wave pinning like behavior - i.e. domain sizes change, but concentrations are stable within domains. This suggests that key features are missing from the simplified model laid out in 2011. This again is not unexpected given the discovery of a number of additional potential feedback circuits not present in these early models (Sailer2015, Lang2023, Bland2024). We have added text to the Results section to stress this feature of the PAR-2 rundowns and this deviation from expectations regarding a wave pinning model.

(2) **Impact of PAR dosage changes on the cue requirements** - A key feature of the model proposed in Goehring et al 2011 and explored further in Khuc Trong 2015 and Gross et al 2019 was that a symmetry-breaking cue destabilizes the unpolarized state, triggering the system to evolve towards a polarized steady-state. So long as the cue is above a threshold, the reaction-diffusion reactions specify the steady state - hence the system is predicted to be robust to reduced cue strength, which is what we see here. What was less obvious in these manuscripts is that this threshold for triggering polarization will be parameter dependent. For example, the magnitude and ratio of aPAR and pPAR dosage can tune this threshold, with low pPAR increasing the threshold cue and high pPAR (or low aPAR) decreasing the threshold. This is likely why *par-2(+/-)* heterozygotes exhibit polarization defects in cue-compromised embryos, while *par-6(+/-)* heterozygotes and partial *par-6(RNAi)* exhibit more ectopic domains (i.e. the system responds to cryptic cues that are normally ignored - see (4) below). We have included additional discussion of the theoretical context to address these points, noting that although the model is likely oversimplified, the qualitative behaviors are nonetheless informative..

(3) **The broader question of model robustness to dosage** - In the model presented in Goehring et al 2011, there is a general sensitivity to dosage with two regimes: one regime in which overall polarity is maintained but the boundary position changes and a second in which aPAR:pPAR ratios exceed permitted ranges leading to loss of polarity. While this is qualitatively similar to what we observe in this work - caveats above notwithstanding - polarity in the Goehring et al (2011) model was only maintained for 25% changes in dosage for the parameters used (note this is highly parameter dependent). Thus, the model appears much more sensitive than the biological system, which again is perhaps not surprising given that the model lacked additional feedback terms which we now know to be present. Measuring the actual sensitivity of the in vivo system was, in fact, a key motivation for this work!

(4) **Boundary position vs ectopic anterior domains** - In the 2011 model, the response of the system to dosage changes was assessed under initial conditions in which we either imposed either initial segregation of aPAR and pPAR proteins or provided a single advection-based cue. What is now clear is that the anterior pole has some intrinsic ability to act as a spatial cue (residual meiotic signals, curvature, or something else) but these "cryptic" cues are usually ignored, presumably because they are normally sub-threshold and/or outcompeted by the normal cue. Depletion of aPARs reduces the threshold for pPAR domains to form, allowing the cryptic anterior cue to trigger PAR-2 domain formation. Thus, in many cases, most of the increased PAR-2 membrane localization in embryos with reduced aPARs is manifest as a second anterior pPAR domain rather than a larger posterior domain, though in both situations the fraction of cortex occupied by PAR-2 increases. Again, we have introduced more discussion of these alternative cues and caveats regarding limits of prior models.

We have now substantially reworked our discussion, which we hope will provide clarity regarding most of these points.

In this sentence, 1D should read 2D: However, embryos heterozygous for par-1, par-3, and pkc-3 did not exhibit such increases (Figure 1D).

Fixed.

Rev. 2:
Rodrigues et al. report the results of a sensitivity analysis examining the effect of reducing PAR protein concentration on worm zygote polarity, asking how downstream pathways function with reduced concentrations. They find a number of phenotypes that are insensitive to changes in Par complex components over some range (that includes the decrease caused by heterozygosity). They first demonstrate that no significant dosage compensation mechanisms are present to account for gene dosage effects, but the amount of PAR on the membrane is affected. They examine zygote asymmetry as a function of PAR levels and find at least some regime where asymmetry is relatively unaffected by changes (not surprisingly at least to the point of the changes caused by heterozygosity). This also holds true for several downstream "outputs", although if the system is sensitized (such as by myosin depletion), it appears the threshold of sensitivity is shifted. Overall the study seems well-executed and the results are clearly communicated

and interesting. However, I found the paper to be very confusing and I hope the authors will consider trying to make the paper more accessible before publication with the following suggestions.

We appreciate the feedback and have reworked the text substantially. We hope that this has resulted in a clearer, more accessible manuscript.

- The author's argue that their data "reveal nonlinear relationships between signal inputs and output responses". Not surprisingly, this conclusion is highly dependent on what one calls an input and output. The authors use PAR concentration as a proxy for PAR output but how do they know that the PAR signal that influences the phenotypes they measure is directly correlated to PAR concentration? For example, aPKC catalytic activity could be the relevant output and might not follow simply from its concentration. The assumption that PAR output and concentration are directly related should be made clear to readers, along with any possible limitations.

We completely agree. Concentration is often taken as equivalent to activity, which is not necessarily the case. At this time we do not have the tools to specifically measure local activity of PAR kinases beyond monitoring the distribution of substrates. We would argue that our data indicates that PAR proteins do respond to changes in dosage of opposing PARs, for example PAR-2 invading the anterior pole as PAR-6 is depleted, and changes in the distribution of PAR-6 to reductions in PAR-2 (note the reductions in domain height and modest expansion). Such results suggest that activity is changing with dosage, though whether this reflects a 1:1 ratio is obviously unclear. Thus, it is certainly possible that the spatial pattern of activity does not strictly match concentration. We would argue that such a mechanism of stabilizing activity profiles to dosage changes would be generally consistent with the central idea of quantitative decoupling between changes in concentration and downstream outputs. Resolving this question will require better methods of monitoring local kinase activity, which unfortunately is beyond the scope of this work. We have added comments on this to the discussion.

- Below "0.25" normalized Par-2 it appears that there is no detectable cortical Par-2 (Fig 4H). How is Par-2 polarized at these levels (Figure 4I)?

Note that asymmetry in Figure 4G and 4I is a composite of the signal-weighted asymmetry of both PAR-2 and PAR-6 as these are dual labeled lines. Hence even if PAR-2 becomes undetectable, as long as PAR-6 asymmetry remains intact, the composite ASI will remain high. We chose this measure to reflect “overall polarity” given the relative contribution of signals from aPAR and pPAR proteins is not clear. We have added additional notation to the text / figure legends to make this more clear.

- The discussion states "One common explanation for robustness to dosage changes is that enzymes are simply present in excess." Why is this "common explanation" introduced so late in the paper and isn't it a possible explanation for the observations?

Perhaps our discussion could have been more clear. Our point was to argue that lack of haploinsufficiency has been attributed to networks in which enzymes are simply present in excess and thus sit on a relatively flat region of the dose-response curve. However, such behavior implies that such systems must generally be insensitive to over-production, which is not naively what one would expect for a system that relies on balance, such as the PAR network. In any case, we have fully reworked our introduction and discussion in the hopes that the narrative is more clear and focused on the core ideas addressed by our work.

- "We reasoned that robustness of developmental processes to changes in gene or protein dosage in a given molecular network could arise from:" Related to the previous point, this enumeration leads the reader to believe that this problem has never been considered before in the literature. But there is an extensive literature, both experimental and theoretical, on this problem that is at least partially touched on in the discussion that would be usefully discussed while motivating possible mechanisms.

We apologize this was unclear. We have rephrased this section and the corresponding part of the introduction to more clearly highlight prior work and link these alternative mechanisms to our specific experiments to test these ideas in the context of PAR polarity.

- I find "quantitative decoupling" to be unnecessarily confusing. Is there a meaningful distinction between quantitative decoupled and the simple and straightforward term "insensitive"?

We agree these are similar. The term “quantitative decoupling” was taken from the literature (Kitano 2004) and emphasizes the lack of quantitative correlation between upstream and downstream modules - i.e. the position of the spindle position is not correlated with PAR protein distributions (e.g. PAR boundary position). We agree that one

could use the term insensitive, but this could also be confusing to some readers as the system is sensitive once depletion approaches 50%. What is the degree of robustness at which a system becomes “insensitive?” We have have rephrased several sections to be more specific in our use this term.

- "Thus, models generally predict that polarization and asymmetric division of the zygote would be sensitive to PAR dosage changes." The authors should be more explicit by what they mean by "sensitive" in these arguments. It's known that some amount of these factors are required for polarity and asymmetric division so at some level they must be sensitive, but do the models insist that there are no regions that are insensitive, as the authors seem to be implying?

Here we primarily intended to raise the potential paradox that polarity generally involves balancing aPAR and pPAR activities, yet heterozygotes have no phenotype. Indeed a key point of the current work is to establish precisely how sensitive the system is to gene/protein dosage changes as this has not been examined in a quantitatively rigorous manner. We have rephrased to make this point more clear and avoided the term “models” here to avoid confusion.

- "Current models for PAR-dependent asymmetric division invoke local concentrations of PAR proteins as the key signals regulating division asymmetry pathways (Galli et al., 2011; Griffin et al., 2011). Yet the phenotypic endpoints of asymmetric division remain wild-type despite significant changes in local PAR concentration. How can we square these observations? " Related to the previous point, I found these statements confusing for two reasons. First, models aren't observations. Second, the question seems like a straw man unless the authors more clearly articulate how the models predict polarity will change with respect to Par concentration.

We again apologize for confusion regarding our use of the term “model” here as it was unclear. We used “model” in a figurative sense in this case meaning that people generally believe that downstream pathways are regulated by local concentrations of PAR kinases. This has been rephrased to avoid confusion.

- "network adaptation - features of the network compensate for imbalance to maintain stable polarity signals, such as changes in feedback strength or pattern; " I was confused as to what the "such as" refers to

This has been rephrased.

- "asymmetric division modules acts to suppress the accumulation of error" The authors should be more specific as to what they mean by "error" and why this error would intrinsically accumulate as they imply (if the error didn't intrinsically accumulate, no action would be required).

This is a valid point and we apologize for being unclear. We have rewritten this section to make our argument more clear.
