# Supplementary material for: Quantitative perturbation-phenotype maps reveal nonlinear responses underlying robustness of PAR-dependent asymmetric cell division
Source: PLoS Biol. 2024 Dec 9;22(12):e3002437. doi: 10.1371/journal.pbio.3002437 (PMC11627365; doi:10.1371/journal.pbio.3002437)
Supplement: Attachment — Submitted filename: PBIOLOGY-D-23-02880R3_ResponseToReviewers.pdf [file pbio.3002437.s005.pdf]

General comments

We thank the editor for their enthusiasm and to the reviewers for constructive comments on our manuscript. In the current revision we have tried to address the final comments raised by the editor and Reviewer 2 and hope the manuscript is now acceptable. Specifically, we have:

- Revised our terminology again to address Reviewer 2's concerns, particularly removing the noted problematic phrases.
- Revised our title to improve clarity.
- Removed citation of "data not shown."
- Deposited relevant data/code into Figshare as requested. A private link for review purposes is available here: https://figshare.com/s/dcee8d4dbf7435559121.

Reviewers' comments

Rev. 1:
I am happy with the changes made to the manuscript in the revision. The inclusion of the spindle severing experiment confirms the results obtained by examining oscillations, and the stability of the PAR-6 boundary upon PAR-2 loss is now clearly explained. I also appreciate the additional discussion of the results in light of previous models. Hence, all my comments have been addressed.

We thank the reviewer for their comments and are glad we were able to address the concerns.

Rev. 2:
My primary criticism of the initial version of the paper was that it was confusing. I appreciate that the authors attempted to revise the paper to make it easier to understand but unfortunately I don't find the revision to be significantly improved in this regard. A good deal of the problem arises from choices that the authors have made to use phrases like "nonlinear readout", "intrinsic robustness", and "quantitative decoupling" (and many more). I specifically noted the latter phrase in my initial review and the authors replied that "The term "quantitative decoupling" was taken from the literature (Kitano 2004)" however I don't believe that they are correct. I didn't find the phrase "quantitative decoupling" in Kitano's robustness review or anywhere else in the literature. In fact, as far as I can tell, there is no difference between that author's usage of "quantitative decoupling" and Kitano's use of "decoupling", with the latter being significantly more straightforward to understand. With that said, I made the same point in my initial review and the authors have revised their paper in the manner that they saw fit so I don't see any reason to delay publication of the paper any further.

We extensively modified the text in response to prior comments and are sorry that the revision did not satisfy the referee. We have further adjusted the terminology to avoid the specific phrases mentioned. Without more information regarding what is meant by "many more," it is difficult to know what else the reviewer feels would improve the manuscript. Finally, it is true that Kitano 2004 did not use *quantitative* decoupling, but we felt this was implied as the concept describes the case in which quantitative variation in inputs (e.g. numbers of molecules or concentrations) is decoupled from the outputs - for example because the system counts oscillations rather than concentration of input signal molecules. We felt this clarification would be helpful to readers, but have adjusted our terminology as requested.
